# Fueling the fire: aspartate deficiency primes and fuels STING activation

**DOI:** 10.1172/JCI206431

**Published:** 2026-06-01

**Authors:** Haitao Jiang, Wenyan Wang, Yang-Xin Fu

**Affiliations:** 1 Department of Basic Medical Sciences, School of Medicine, and; 2 State Key Laboratory of Molecular Oncology, Tsinghua University, Beijing, China.; 3 Changping Laboratory, Beijing, China.

## Abstract

Cytosolic DNA sensing through the cyclic GMP-AMP synthase (cGAS)–stimulator of interferon genes (STING) pathway has emerged as a promising strategy to elicit antitumor immunity. However, clinical translation of STING agonists has been hindered by limited efficacy and dose-limiting inflammatory toxicity, highlighting that simply providing activating ligands is insufficient to achieve durable immune responses. In this issue of the *Journal of Clinical Investigation*, Liao et al. showed that intracellular aspartate availability critically shapes STING signaling responsiveness. Aspartate deficiency disrupted pyrimidine synthesis, induced mtDNA stress, and engaged a feed-forward Z-DNA binding protein 1 and receptor interacting serine/threonine kinase 1/3 axis. Rather than directly triggering immunity, this metabolic state primed DNA sensing and fueled downstream signaling, thereby enabling robust and sustained antitumor immune responses. Together, these findings position nucleotide metabolism as a key determinant of innate immune responsiveness and suggest that metabolic conditioning may enhance the efficacy of STING-targeted therapies.

## Metabolic gating of DNA sensing limits STING therapy

The cyclic GMP-AMP synthase (cGAS)–stimulator of interferon genes (STING) pathway has emerged as a central driver of antitumor immunity, linking cytosolic DNA sensing to type I IFN production and T cell priming (1). These properties have driven efforts to therapeutically activate STING using synthetic agonists, radiotherapy, or DNA-damaging chemotherapy. Yet, clinical responses have been modest, often limited by transient signaling, tumor-intrinsic resistance, and dose-limiting inflammatory toxicity (2, 3).

These challenges suggest that STING activation is not constrained merely by ligand availability but also by intrinsic regulatory mechanisms. Increasing evidence points to cellular metabolism as a key determinant of innate immune responsiveness. Metabolic pathways, including lipid metabolism, mitochondrial function, and amino acid availability, shape DNA sensing at multiple levels, from ligand generation to downstream signaling (4–6). In this framework, metabolism functions as a gatekeeper, determining whether DNA-sensing signals remain subthreshold or become amplified.

Liao et al. have extended this concept by identifying aspartate metabolism as a critical regulator of STING responsiveness (7). A screen of small molecule metabolic inhibitors in multiple cell lines identified that a broad inhibitor of aminotransferases, aminooxyacetic acid (AOA), exerted profound regulation over cGAS/STING signaling. AOA treatment induces aspartate depletion in cells, as it inhibits glutamate oxaloacetate transaminase 2 (GOT2) and glutamate pyruvate transaminase 2 (GPT2), two aminotransferases that are essential for aspartate synthesis. Their findings suggest that metabolic state shapes the magnitude and propagation of innate immune signaling, providing a mechanistic explanation for the limited efficacy of STING agonists as monotherapies.

## Aspartate depletion amplifies, rather than triggers, STING activation

**Figure 1:**
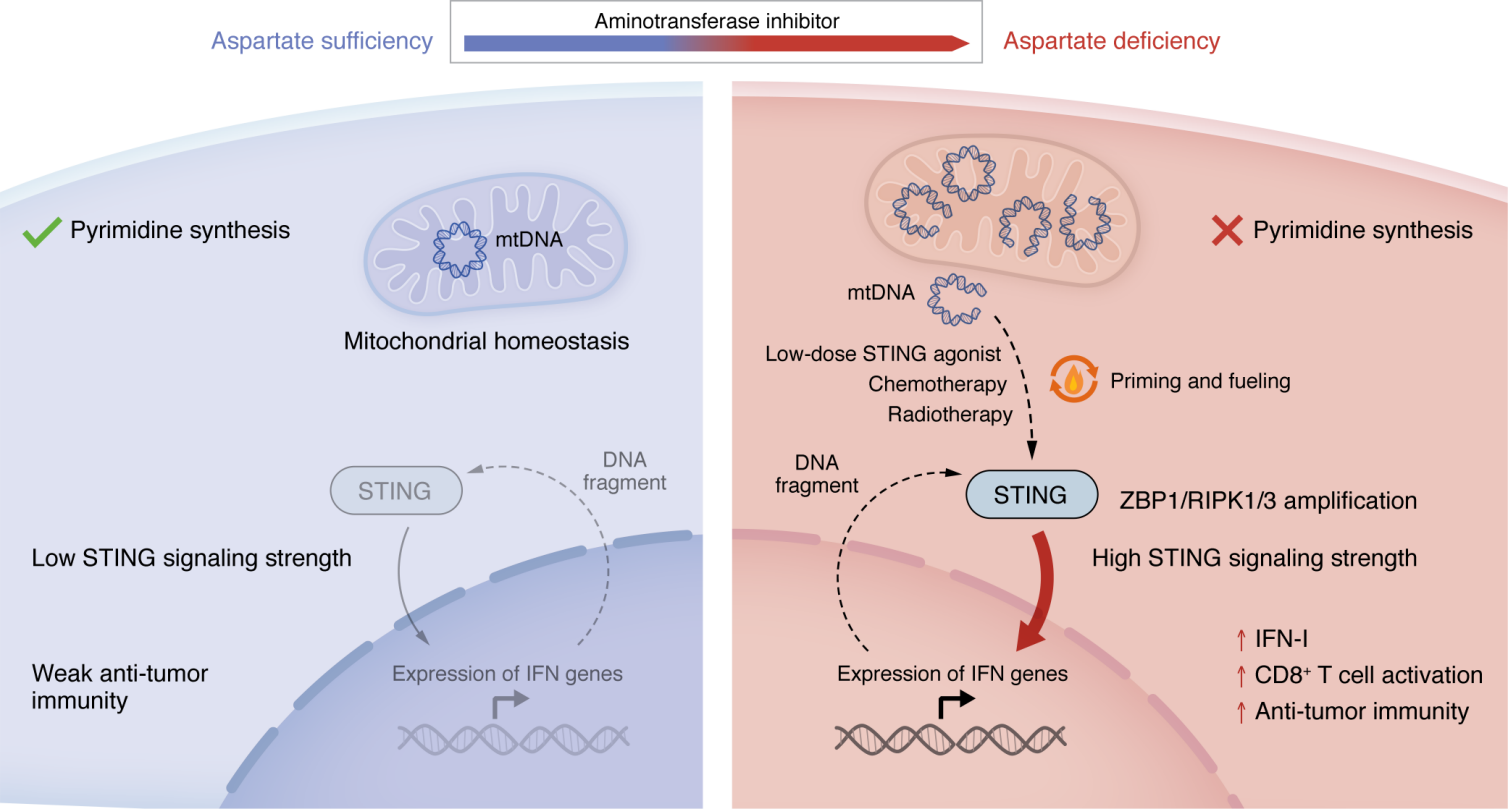
Aspartate metabolism primes and fuels STING-mediated antitumor immunity. (Left) In an aspartate-sufficient state, de novo pyrimidine synthesis is efficient, preserving mitochondrial DNA (mtDNA) integrity within mitochondria. Minimal mtDNA is released into the cytosol, resulting in weak cGAS/STING pathway activation, limited type I interferon (IFN-I) production, and insufficient CD8^+^ T cell activation, ultimately leading to ineffective antitumor immunity. (Right) Aspartate deficiency impairs pyrimidine synthesis, causing nucleotide scarcity that destabilizes mtDNA and promotes its cytosolic release. Cytosolic mtDNA potentiates STING signaling and enables a feed-forward amplification circuit through the ZBP1–RIPK1/3 axis. This sustained signaling intensifies IFN-I production and CD8^+^ T cell–mediated antitumor immunity, thereby converting otherwise weak stimuli (low-dose STING agonists) or transient stimuli (radiotherapy and chemotherapy) into robust and sustained antitumor immune responses.

A key conceptual advance of this study is the distinction between activation and sensitization. Across distinct cell lines, AOA induced only modest IFN responses but markedly amplified signaling in response to STING agonists, chemotherapy, or radiotherapy (7) (Figure 1). This observation indicates that aspartate metabolism operates upstream of canonical signaling and functions as a priming mechanism rather than a direct trigger.

This “licensing” model aligns with broader observations in innate immunity. Subthreshold stresses — including replication stress, chromatin instability, and mitochondrial dysfunction — can generate low levels of cytosolic DNA that are insufficient to activate cGAS-STING alone but enhance responsiveness to secondary stimuli (8, 9). In this context, aspartate availability acts as a metabolic rheostat that tunes DNA-sensing sensitivity.

Mechanistically, by lowering intracellular aspartate via inhibition of GOT2 and GPT2, AOA treatment impaired de novo pyrimidine synthesis through the carbamoyl-phosphate synthetase 2/aspartate transcarbamylase/dihydroorotase (CAD) complex and led to nucleotide insufficiency. Pyrimidine supplementation reversed this effect, identifying nucleotide scarcity as a key driver (7). This coupling suggests that nucleotide limitation is sensed as cellular stress, thereby augmenting the magnitude of immune activation in response to DNA-sensing signals. In tumors, where metabolic rewiring is pervasive, this mechanism may critically shape responsiveness to immunotherapy.

## mtDNA stress engages a feed-forward amplification circuit

Aspartate deficiency links metabolic stress to sustained STING activation by coordinating both ligand generation and signaling amplification. By limiting pyrimidine synthesis, aspartate depletion selectively destabilized mtDNA and promoted its voltage-dependent anion channel–dependent release, providing a priming signal for cGAS-STING activation. In parallel, Z-DNA binding protein 1 (ZBP1) induction enabled receptor interacting serine/threonine kinase 1/3–dependent (RIPK1/3-dependent) phosphorylation of interferon regulatory factor 3 (IRF3), prolonging IFN signaling independently of TANK binding kinase 1 (TBK1) and establishing a feed-forward circuit (7).

This mechanism contrasts with prior studies showing that ZBP1 enhances radiotherapy-induced STING activation through mixed lineage kinase domain like pseudokinase–dependent necroptosis, wherein inflammatory cell death drives mtDNA release (10). Liao et al. (7) reported that ZBP1 instead functions independently of necroptosis, highlighting its context-dependent role as a signaling amplifier. More broadly, Liao et al.’s findings align with emerging evidence that innate sensing pathways, including RIG-I/MAVS (11) and TLR signaling (12), can potentiate STING activation through mitochondrial stress or pathway crosstalk. Together, these findings position STING as a hub within an integrated innate immune network shaped by metabolic state.

## Implications for cancer therapy

The therapeutic implications of Liao et al.’s work (7) are substantial. By enhancing STING signaling output, aspartate depletion sensitized mouse tumors to otherwise weak or transient stimuli, enabling robust responses to low-dose STING agonists as well as to chemotherapy and radiotherapy, which generate DNA ligands but often do not trigger sustained signaling.

This study reframes current strategies for STING-based therapy. Rather than simply increasing agonist potency, it may be necessary to modulate tumor metabolism to permit effective signaling. Similar principles have emerged in other areas of immuno-oncology, where metabolic interventions enhance immune checkpoint blockade or T cell function (13, 14).

Importantly, the effect of aspartate depletion appears largely tumor cell intrinsic, with minimal direct impact on CD8^+^ T cells. This suggests a potential therapeutic window in which tumor metabolism can be selectively targeted to amplify antitumor immunity.

## Broader implications: metabolism and inflammatory disease

While metabolic priming may enhance antitumor immunity, heightened DNA-sensing activity could have unintended consequences. The cGAS/STING pathway is a central driver of sterile inflammation, aging, and autoimmune disease (15, 16), where chronic activation that is often triggered by mitochondrial dysfunction or genomic instability leads to sustained IFN production.

Aspartate metabolism may represent a previously underappreciated link between mitochondrial function and inflammatory signaling. Recent work has identified mitochondrial aspartate production as a key metabolic determinant of inflammatory output, supporting TNF biogenesis and cytokine expression in immune cells and thereby promoting autoimmune tissue inflammation (17). Reduced aspartate availability could promote mtDNA instability and cytosolic DNA accumulation, providing a metabolic mechanism linking mitochondrial stress to aberrant innate immune activation. The DNA/IFN/inflammation/autoimmune pathway has strong clinical associations.

These findings highlight an important trade-off. While transient reinforcement of STING signaling may benefit cancer therapy, sustained or systemic perturbation of aspartate metabolism could exacerbate inflammatory pathology. More broadly, this work underscores a central principle: metabolic state not only supports immune responses but also modulates their intensity and downstream consequences.

## Conclusions and future directions

Liao et al. identified aspartate metabolism as a key determinant of innate immune sensitivity, revealing that metabolic state enhances the signaling output of the cGAS/STING pathway. By linking nucleotide availability to mitochondrial genome stability and feed-forward signaling, this work establishes a conceptual framework for how metabolic stress augments the DNA-sensing pathway. This framework further suggests that differential allocation of nucleotide pools between mitochondrial and nuclear compartments may shape genome stability and immune activation. Moreover, the identification of a ZBP1–RIPK1/3–IRF3 axis expands the signaling architecture downstream of STING, providing a foundation for understanding how canonical and noncanonical kinases coordinate IFN responses across diverse cellular contexts and therapeutic settings.

Future work should define how aspartate metabolism is regulated within the tumor microenvironment, where nutrient availability, hypoxia, and metabolic competition shape intracellular aspartate pools and drive heterogeneous immune responsiveness. In this setting, tumor cell aspartate metabolism may serve as a predictive biomarker of responsiveness to STING-based therapies. Integrating metabolomic profiling, isotope tracing, and expression of key metabolic enzymes — including GOT2, GPT2, and CAD — could support patient stratification and identify tumors most likely to benefit from STING agonists or DNA-damaging therapies.

More broadly, these findings highlight a central principle: innate immune activation is governed not only by danger signals but also by the metabolic context in which they are sensed. Defining how metabolic states tune immune thresholds may enable both more precise cancer immunotherapy and improved control of inflammatory disease.

## Conflict of interest

The authors have declared that no conflict of interest exists.

## Funding support

- National Natural Science Foundation of China (32370967 to WW).

## Version 1 06/01/2026

Electronic publication
